# Tolerability for older, persistence for younger: a real-world evidence on sacubitril/valsartan in an Asian heart failure cohort across age

**DOI:** 10.3389/fcvm.2025.1620266

**Published:** 2025-07-22

**Authors:** Po-Kai Chan, Chu-Yu Hsu, Chao-Chin Lee, Fan-Han Yu, Fa-Po Chung, Chia-Te Liao, Jin-Long Huang, Huai-Wen Liang, Ying-Hsiang Lee, Po-Lin Lin, Wei-Ru Chiou, Chien-Yi Hsu, Hung-Yu Chang, Wen-Yu Lin

**Affiliations:** ^1^ School of Medicine, National Defense Medical Center, Taipei, Taiwan; ^2^ Division of Cardiology, Department of Medicine, Tri-Service General Hospital, National Defense Medical Center, Taipei, Taiwan; ^3^ Division of Cardiology, Department of Medicine, Taoyuan Armed Forces General Hospital, Taoyuan, Taiwan; ^4^ Divsion of Cardiology, Department of Medicine, Taipei Veterans General Hospital, Taipei, Taiwan; ^5^ Faculty of Medicine, School of Medicine, National Yang Ming Chiao Tung University, Taipei, Taiwan; ^6^ Divsion of Cardiology, Chi-Mei Medical Center, Tainan, Taiwan; ^7^ Department of Public Health, College of Medicine, National Cheng Kung University, Tainan, Taiwan; ^8^ Cardiovascular Center, Taichung Veterans General Hospital, Taichung, Taiwan; ^9^ Division of Cardiology, Department of Internal Medicine, E-Da Hospital, I-Shou University, Kaohsiung, Taiwan; ^10^ Department of Medicine, MacKay Medical College, New Taipei City, Taiwan; ^11^ Cardiovascular Medicine, MacKay Memorial Hospital, Taipei, Taiwan; ^12^ Department of Artificial Intelligence and Medical Application, MacKay Junior College of Medicine, Nursing, and Management, Taipei, Taiwan; ^13^ Divsion of Cardiology, Department of Internal Medicine, Hsinchu MacKay Memorial Hospital, Hsinchu, Taiwan; ^14^ Department of Nursing, MacKay Junior College of Medicine, Nursing, and Management, Taipei, Taiwan; ^15^ Divsion of Cardiology, Department of Internal Medicine, Taitung MacKay Memorial Hospital, Taitung, Taiwan; ^16^ College of Science and Engineering, National Taitung University, Taitung, Taiwan; ^17^ Divsion of Cardiology and Cardiovascular Research Center, Department of Internal Medicine, Taipei Medical University Hospital, Taipei, Taiwan; ^18^ Division of Cardiology, Department of Internal Medicine, School of Medicine, College of Medicine, Taipei Heart Institute, Taipei Medical University, Taipei, Taiwan; ^19^ Heart Center, Cheng Hsin General Hospital, Taipei, Taiwan

**Keywords:** sacubitril/valsartan, age, tolerability, heart failure, real-world study, older patient

## Abstract

**Background:**

Sacubitril/Valsartan (S/V) benefits patients with heart failure with reduced ejection fraction (HFrEF), but its tolerability and clinical outcomes across age groups, especially the elderly, remain unclear. This real-world study evaluates these factors in an Asian cohort.

**Methods:**

This retrospective cohort study analyzed data from the Treatment with Angiotensin Receptor Neprilysin Inhibitor for Taiwan Heart Failure Patients (TAROT-HF) registry (2017–2018). Patients were stratified into three age groups: <65, 65–74, and ≥75 years. Tolerability was defined as achieving at least 50% of the target S/V dosage (200 mg/day). Baseline characteristics, treatment doses, and clinical outcomes—including the composite of first unplanned heart failure hospitalization (HFH) or cardiovascular (CV) death, all-cause mortality, CV death, and HFH—were assessed over 5 years.

**Results:**

Among 1,987 patients, older adults had more comorbidities and received lower S/V doses. Achieving tolerability significantly reduced composite outcome risk in patients <65 (HR = 0.40, 95% CI: 0.27–0.59, *p* < 0.001), all-cause mortality (HR = 0.30, *p* < 0.001), CV death (HR = 0.41, 95% CI: 0.21–0.80, *p* = 0.009), and HFH (HR = 0.41, 95% CI: 0.27–0.62, *p* < 0.001). Those aged 65–74 had similar benefits except for CV death. In patients ≥75, reaching tolerability improved composite outcome (HR = 0.60, 95% CI: 0.39–0.91, *p* = 0.017) and HFH (HR = 0.60, 95% CI: 0.38–0.95, *p* = 0.029). Partial dosing still provided protection in younger patients.

**Conclusion:**

S/V improves HFrEF clinical outcomes across age groups in an Asian population, especially when achieving tolerability, defined as reaching ≥50% of the target dose. While this association was less pronounced in older patients, our result suggested that individualized dosing strategies should prioritize persistence in younger patients while accommodating tolerability in older populations.

## 1 Introduction

The prevalence of heart failure (HF) in a general population increases with age, from 1% among those aged 45–55 years to 10% among those aged 80 years and more (1, 2). HF is a major cause of mortality, morbidity, and hospitalization in older adults. Sacubitril/Valsartan (S/V), a novel combination drug containing an angiotensin receptor blocker (Valsartan) and a neprilysin inhibitor (Sacubitril), or angiotensin receptor neprilysin inhibitors (ARNi), had been approved for the cornerstone treatment of patients with HF with reduced ejection fraction (HFrEF). The PARADIGM-HF study demonstrated the superiority of S/V (200 mg bid) over enalapril (10 mg bid) in reducing mortality and morbidity in chronic HFrEF patients (3). A *post-hoc* analysis of the PARADIGM-HF trial demonstrated that S/V was more beneficial than enalapril across the spectrum of age (4). In the PIONEER-HF study, which enrolled a significant proportion of patients aged 65 and older, individuals hospitalized for acute decompensated heart failure with reduced ejection fraction experienced a more substantial reduction in N-terminal pro-brain natriuretic peptide (NT-proBNP) concentration with the initiation of S/V therapy compared to enalapril therapy (5). Nevertheless, the PARADIGM-HF trial excludes those who couldn't tolerate the treatment doses during the running period, and only about half of the patients in the S/V treatment group reached the target dose in the PIONEER-HF trial. Older HF patients are undoubtedly at higher risk of adverse effects during standard HF management than younger patients (6). There is no clear and evident treatment suggestion in the older population, and withdrawal or failure to reach the target dose of S/V has become a frequent clinical issue (7). Therapeutic decisions are often based on the clinical experience of physicians and extrapolation of data from clinical trials that lack adequate representation of old and very old subjects (8). Little is known about the dosage and tolerability of S/V across the older age category, especially in Asian population.

The objective of this study is twofold: first, to elucidate the clinical characteristics of older adults with HFrEF treated with S/V in Taiwan; second, to assess the utilization and tolerability of S/V across different age categories, with the aim of evaluating both the tolerability of the drug and the therapeutic benefits in the real-world clinical practice.

## 2 Methods

### 2.1 Study design and patient population

The present study extracted and analyzed data from the TAROT-HF (Treatment with Angiotensin Receptor neprilysin inhibitor fOr Taiwan Heart Failure patients) cohort, a principal investigator-initiated multicenter, retrospective, and observational study in Taiwan, that enrolled chronic symptomatic HFrEF outpatients and those hospitalized due to acute decompensated HFrEF patients from 10 hospital between 2017 and 2018 (9). Comprehensive clinical data for all patients were meticulously recorded by dedicated research assistants in the TAROT-HF study. Baseline data collection encompassed various essential parameters, including age, gender, body mass index (BMI), etiologies of heart failure, New York Heart Association (NYHA) functional class, underlying comorbidities, the utilization of cardiac devices, concomitant medications, electrocardiographic findings, estimated glomerular filtration rate (eGFR), and other laboratory results. Additionally, the initial and the maintenance S/V dosage prescribed to each patient was recorded, and physicians made clinical decisions regarding dosage titration during follow-up based on individual patient presentations.

For current study, the inclusion criteria comprised individuals age above 18 with HFrEF (echocardiographic LVEF documented as ≤40%), and initiation of S/V treatment at any dosage. Subsequently, patients included were divided into three age groups: <65, 65–74, and ≥75 year. The primary objectives of treatment tolerability were analyzed for each age group, as were the secondary objectives of clinical outcomes.

### 2.2 Outcome definition

#### 2.2.1 Tolerability

There is no standard definition of tolerability. In the TITRATION study, tolerability success was defined as maintenance of the target dose for at least the final 2 weeks prior to study completion, while treatment success referred to achieving the target dose of S/V without down-titration or dose interruption over 12 weeks (10). In the TRANSITION study, tolerability was defined as any dose of S/V within 10 weeks (11).

To investigate the impact of age on S/V tolerability, particularly in the older adults within our study, we defined tolerability as the ability to withstand at least 50% of the target S/V dosage (100 mg or 200 mg twice daily) for the final 6 months of follow-up, regardless of previous dose interruption or down-titration. This threshold was selected based on evidence from a *post-hoc* analysis of PARADIGM-HF, which showed that the benefits of S/V were maintained at lower-than-target doses, but not in patients receiving less than 50% of the target dose (12). Meanwhile, in contrast to the relatively short durations used in prior studies (TITRATION, TRANSITION), our definition aimed to capture tolerability under real-world, long-term treatment conditions.

Treatment outcomes were categorized into three groups: (1) the discontinuation group, (2) sustained users who achieved at least 50% of the target sacubitril/valsartan (S/V) dose (defined as tolerability), and (3) sustained users who continued therapy without achieving tolerability. Patients with temporary interruptions were classified as sustained users if they resumed therapy during the final six months of the follow-up period. Reasons for discontinuation were also recorded.

#### 2.2.2 Clinical endpoint

Over a follow-up period, each patient was followed retrospectively by specialized research assistants to assess the incidence of the composite of cardiovascular death and first unplanned heart failure hospitalization (HFH), all-cause mortality, cardiovascular death only, and first unplanned HFH, respectively. The first HFH is defined as admission due to heart failure after S/V initiation.

### 2.3 Statistical analysis

Descriptive summaries were presented for all patients and for subgroups of patients. The continuous variables were presented as the mean value with standard deviation, and the categorical variables were shown as numbers and percentages. The baseline characteristics had over 95% data completeness across variables, with missing values handled using complete case analysis. Outcome data (including treatment continuation and dosing) were available for all patients included in the final analysis, ensuring robustness of the reported findings. The differences for continuous variables were tested using the student's *t*-test or the Wilcoxon rank-sum test. The *X*^2^ test or Fischer's exact test was used to examine the comparisons between categorical variables. The survival analysis was conducted using the Kaplan–Meier method and compared using the log-rank test to estimate the time-to-event data, providing a visual representation of the survival experience of the cohorts. To account for baseline differences among the study groups and ensure a more accurate estimation of the intervention effects, a Cox proportional hazards regression model was developed. The variables chosen were based on their clinical relevance and previous literature known to influence the outcomes. The confounding factors included in the adjustment were history of stroke, atrial fibrillation, previous HFH, hypertension (HTN), diabetes mellitus (DM), baseline BMI, eGFR, systolic blood pressure (SBP) and left ventricular ejection fraction (LVEF). A forest plot was generated based on this multivariable Cox model to visualize the hazard ratio (HR) and confidence intervals (CI) of S/V use across different age groups. If key variables had excessive missingness, those cases were excluded from the analysis. Patients lost to follow-up, if any, were compared with those who completed follow-up to identify any significant differences in baseline characteristics. Additionally, sensitivity analyses were performed to assess the robustness of the findings. A *P*-value of <0.05 was considered statistically significant. The statistical analyses were performed using IBM SPSS Statistics 24.0 software (IBM SPSS IBM Corp, Armonk, NY, USA), and programming language R (R Core Team, 2022) with remote package “survminer”, “survival”, and “forestplot” for survival analysis and plotting. Sensitivity test was conducted by eliminating those who temporarily discontinued the S/V during the follow-up period.

## 3 Results

### 3.1 Baseline demographic and clinical characteristics

A total of 1,987 patients were included in the analysis with the average following up time for 952 days. No patient was missing or lost following up. Overall, 1,106 (55.7%), 445 (22.4%), and 436 (21.9%) patients with age of <65, 65–74, and ≥75 years old, respectively. Baseline characteristics of patients, stratified by age, were presented in Table 1. Generally, comparing to the younger age group, patients with aged ≥75 had higher proportion of male gender (62.4%), hypertension (61.5%), atrial fibrillation (44.7%), and NYHA class III–IV (39.0%) at screening. In addition, the older patients had lower BMI (23.6 ± 3.9) and eGFR (51.0 ± 23.1). Regarding the concomitant medication at baseline, the proportion of combined S/V and beta-blocker, mineralocorticoid receptor antagonist, and ivabradine declined with increasing age.

**Table 1:** Baseline characteristics of patients, stratified by age.

Item	Unit	Age group (years)			All	*p* value
		Below 65	65–75	Above 75		
		(*N* = 1,106)	(*N* = 445)	(*N* = 436)	(*N* = 1,987)	
Baseline characteristics and comorbidities						
BMI	mean (SD)	26.8 (5.2)	24.6 (4)	23.6 (3.9)	25.6 (4.9)	.000
Gender (Female)	*n* (%)	192 (17.4)	113 (25.4)	164 (37.6)	469 (23.6)	.000
Hypertension	*n* (%)	393 (35.5)	254 (57.1)	268 (61.5)	1,041 (52.4)	.000
Diabetes mellitus	*n* (%)	519 (46.9)	203 (45.6)	196 (45)	792 (39.9)	.000
Prior MI	*n* (%)	275 (24.9)	146 (32.8)	140 (32.1)	561 (28.2)	.001
Prior stroke	*n* (%)	90 (8.1)	66 (14.8)	60 (13.8)	216 (10.9)	.000
Atrial fibrillation	*n* (%)	284 (25.7)	165 (37.1)	195 (44.7)	644 (32.4)	.000
Dyslipidemia	*n* (%)	526 (47.6)	206 (46.3)	192 (44)	924 (46.5)	.456
Prior HFH	*n* (%)	635 (57.4)	290 (65.2)	284 (65.1)	1,209 (60.8)	.002
SBP	mean (SD), mmHg	122.2 (20.6)	120.8 (18.5)	120.9 (19.9)	121.6 (20)	.347
eGFR	mean (SD), ml/min/1.732	73.2 (34.7)	57.4 (25.5)	51 (23.1)	64.8 (32)	.000
NYHA						.024
I–II	*n* (%)	754 (68.2)	300 (67.4)	266 (61)	1,320 (66.4)	
III–IV	*n* (%)	352 (31.8)	145 (32.6)	170 (39)	667 (33.6)	
LVEF (%)	mean (SD)	27.5 (6.5)	28.5 (6.2)	29.3 (5.8)	28.1 (6.3)	.000
LA diameter	mean (SD), mm	46.8 (9)	44.7 (8.4)	45.3 (9.4)	46 (9)	.000
RVSP	mean (SD), mmHg	38.1 (15)	40.4 (15.4)	42 (21.8)	39.5 (16.9)	.000
Baseline medications use						
RAASi	*n* (%)	816 (76.3)	319 (74.2)	297 (70.7)	1,432 (74.6)	.084
ACEi	*n* (%)	188 (17.6)	71 (16.5)	67 (16)	326 (17)	.724
ARB	*n* (%)	629 (58.8)	252 (58.6)	228 (54.3)	1,109 (57.8)	.264
BB	*n* (%)	929 (84.1)	347 (78)	309 (70.9)	1,585 (79.8)	.000
MRA	*n* (%)	761 (68.8)	274 (61.6)	251 (57.7)	1,286 (64.8)	.000
Diuretics	*n* (%)	677 (61.2)	247 (55.5)	276 (63.3)	1,200 (60.4)	.043

BMI, body mass index; MI, myocardial infarction; HFH, heart failure hospitalization; SBP, systolic blood pressure; eGFR, estimated glomerular filtration rate; NYHA, New York heart association; LVEF, left ventricular ejection fraction; LA, left atrium; RVSP, right ventricular systolic pressure; RAASi, renin-angiotensin aldosterone system inhibitors; ACEi, angiotensin-converting enzyme inhibitors; ARB, angiotensin-receptor blockers; BB, beta-blocker; MRA, mineralocorticoid receptor antagonist; SD, standard deviation.

### 3.2 Tolerability

Table 2 demonstrated the initial and achieved daily dosage of S/V and the proportion of tolerability in different age groups. The initial dosage of S/V prescription was lowest in the oldest age group with 102.6 ± 50.9 mg daily and the dosage increased when age declined. In addition, patients aged ≥75 had the lowest achieved daily dosage (137.3 ± 90.56 mg) compared with other age groups. Discontinuation rate was 10.6% in overall population, whereas the youngest group exhibited a significantly lower failure rate (8.0%, *p* < 0.001).

**Table 2:** Dosages and proportion of tolerability and treatment success, stratified by age.

Item	Unit	Age group (years)			All	*p* value
		Below 65	65–75	Above 75		
		(*N* = 1,106)	(*N* = 445)	(*N* = 436)	(*N* = 1,987)	
Follow up duration	mean (SD), day	993.66 (363.6)	948.93 (383.18)	850.23 (410.77)	952.17 (382.86)	.000
Sacubitril/valsartan dose						
Initial daily dose	mean (SD), mg	116.8 (62.3)	107.1 (54)	102.6 (50.9)	111.5 (58.5)	.000
Achieved daily dose	mean (SD), mg	165.75 (96.05)	150.28 (89.05)	137.3 (90.56)	156.05 (94.02)	.000
Treatment result						
Discontinued	*n* (%)	88 (8)	55 (12.4)	68 (15.6)	211 (10.6)	.000
<50% target dose	*n* (%)	474 (42.9)	210 (47.2)	221 (50.7)	905 (45.5)	.000
≥50% target dose	*n* (%)	544 (49.2)	180 (40.4)	147 (33.7)	871 (43.8)	.000
Reach target dose	*n* (%)	104 (9.4)	34 (7.6)	28 (6.4)	162 (8.2)	.000

Nevertheless, in the elder patients, the percentage of patients achieving ≥50% of the target dosage declined. The tolerability was highest (48.1%) in patients younger than 65 years old, followed by 39.1% in those aged 65–74 and the patients over 75-year-old had the lowest proportion of tolerability of 33.3%. The reasons for discontinuation of S/V, stratified by age, were demonstrated in Table 3. Overall, the most common side effect was hypotension (39.3%), following by renal function impairment or hyperkalemia (20.4%), and allergy (7.6%). In the oldest population, the main reasons for discontinuation were symptomatic hypotension (36.8%), which is similar to the younger populations; however, S/V discontinuation due to renal function impairment (or hyperkalemia) was significantly higher in the oldest age group (27.9%, *p* = 0.004).

**Table 3:** The reasons for discontinuation of Sac/Val, stratified by age.

Reason	Group	Age group (years)			All	*p* value
		Below 65	65–75	Above 75		
		(*N* = 88)	(*N* = 55)	(*N* = 68)	(*N* = 211)	
Hypotension	*n* (%)	31 (35.2)	27 (49.1)	25 (36.8)	83 (39.3)	.116
Renal dysfunction or hyperkalemia	*n* (%)	12 (13.6)	12 (21.8)	19 (27.9)	43 (20.4)	.004
Allergy	*n* (%)	7 (8)	2 (3.6)	7 (10.3)	16 (7.6)	.132
Other reasons	*n* (%)	37 (42)	15 (27.3)	17 (25)	69 (32.7)	.161

### 3.3 Clinical outcomes

**Figure 1:**
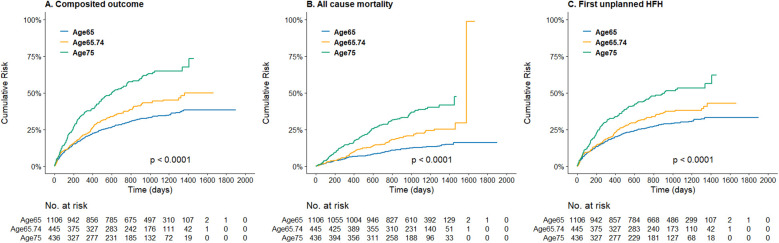
Kaplan–meier curve of **(A)** the composite incidence of cardiovascular death or first unplanned heart failure hospitalization, **(B)** the all-cause mortality, and **(C)** the first unplanned heart failure hospitalization, stratified by age groups.

**Figure 2:**
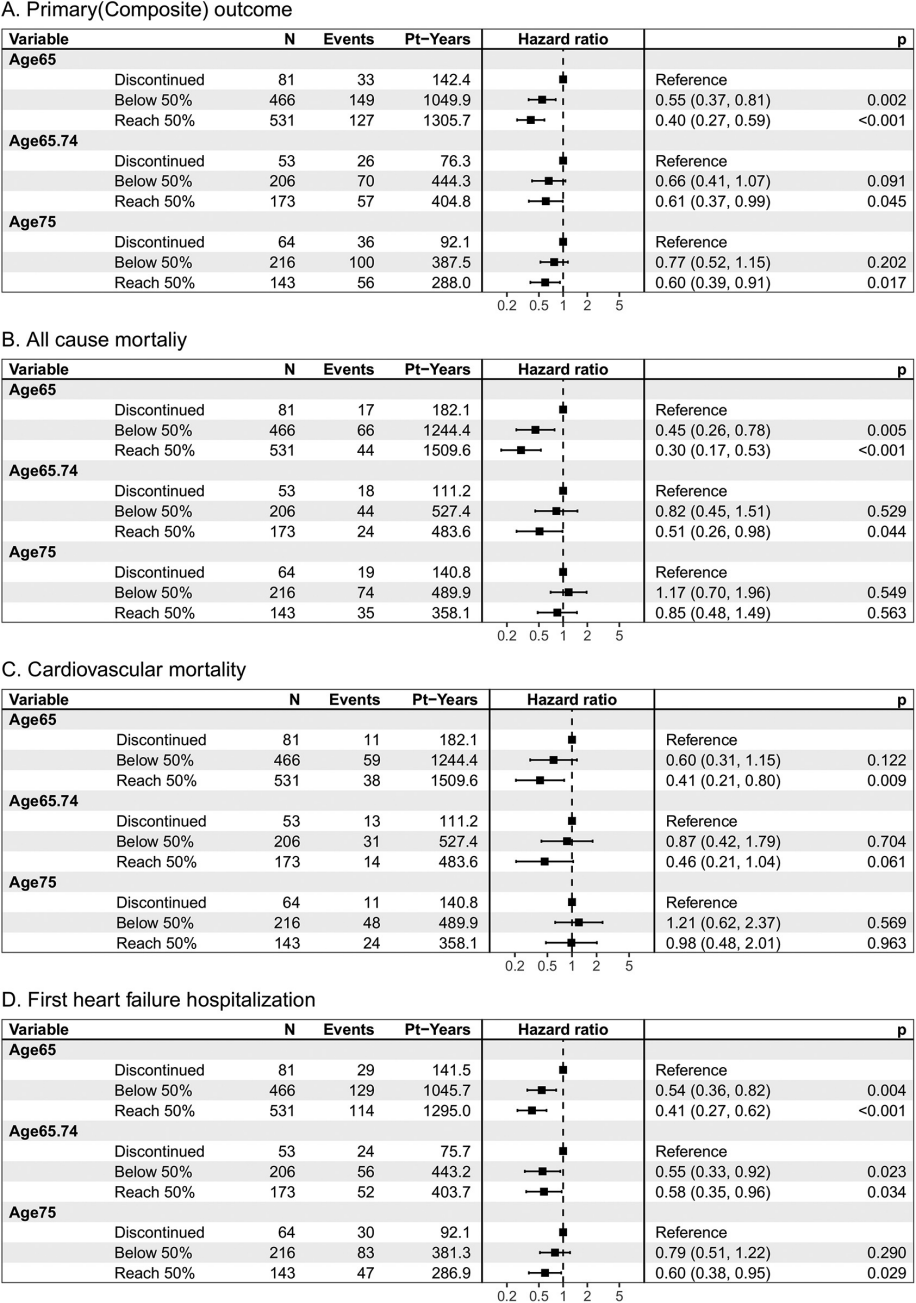
Clinical outcomes of **(A)** the composite incidence of cardiovascular death or first unplanned heart failure hospitalization, **(B)** the all-cause mortality, and **(C)** cardiovascular death, and **(D)** the first unplanned heart failure hospitalization, by age category and treatment response. Pt-Years, patient years p, *p value*. N, number of patients.

The Figure 1 demonstrated the cumulative incidence curves of clinical endpoints of study patients stratified by age. Advanced age was associated with a significantly higher risk of composite endpoints including cardiovascular death and first unplanned HFH, as well as all-cause mortality, and fist unplanned HFH. The multivariable Cox analysis was used to present the hazard difference in each age group based on achieving tolerability or not, as shown in Figure 2. For individuals aged 65 and younger, reaching tolerability substantially reduces the risk of the composite outcome of cardiovascular death and first unplanned HFH (HR = 0.40, 95% CI: 0.27–0.59, *p* < 0.001), as well as all-cause mortality (HR = 0.30, 95% CI: 0.17–0.53, *p* < 0.001), cardiovascular death (HR = 0.41, 95% CI: 0.21–0.80, *p* = 0.009), and first unplanned HFH (HR = 0.41, 95% CI: 0.27–0.62, *p* < 0.001) compared to those who discontinued the intervention. Similarly, those aged 65–74 who reached the 50% target also experienced a significant reduction in these outcomes except for cardiovascular death (HR = 0.46, 95% CI: 0.21–1.04, *p* = 0.061). Notably, in the oldest age group reaching tolerability, despite less pronounced, the benefits of the S/V were observed for the composite outcome (HR = 0.60, 95% CI: 0.39–0.91, *p* = 0.017) and HFH (HR = 0.60, 95% CI: 0.38–0.95, *p* = 0.029). Furthermore, those who didn't reach tolerability nor discontinue had a lower risk than those who stopped S/V in the youngest age group, except for cardiovascular death (HR = 0.60, 95% CI: 0.31–1.15, *p* = 0.122), indicating that some intervention still protects against adverse cardiovascular outcomes in this population. Sensitivtiy test showed similar results, which is showed in supplements (Supplementary Figure).

## 4 Discussion

The study delves into an extensive examination of the tolerability and clinical outcomes associated with S/V in patients with HFrEF across various age groups within the TAROT-HF cohort. Within our cohort, the older subgroup, particularly those aged 75 and above, exhibited distinctive characteristics with more comorbidity and concomitant medication. In addition, despite a decline in the achieved daily dose of S/V with increasing age, a notable finding was their tolerance to any S/V dose comparable to their younger counterparts. Another observation that emerged from our analysis was that patients who achieved at least 50% of the target daily dose of S/V appeared to have better clinical outcomes despite not being significant in older adults. Additionally, the data suggested that even if patients cannot reach the 50% intervention target, avoiding discontinuation entirely in certain age groups may still be worthwhile.

It's worth noting that clinical trials often exclude or under-represent older populations, raising concerns about the external validity of their results. Nevertheless, S/V has demonstrated survival benefits and improvements in physical and social activity, even among older adults and multi-morbid patients (13–19). Our study aligns with previous research, such as a *post-hoc* analysis of PARADIGM-HF, indicating that the benefits of S/V relative to enalapril were maintained even at lower-than-target doses, despite individuals taking less than 50% of the target dose (daily dose 200 mg) showing insignificant results (12). In a *post-hoc* analysis of PROVE-HF study, even individuals receiving S/V at a low dose had shown significant improvement in cardiac stress biomarkers, health status, and clinically meaningful reverse cardiac remodeling (20). Growing real-world evidence in Asian countries also suggested the effectiveness of S/V with improvements of cardiac function or markers, despite lower initial and maintenance doses, among patients in Japan and China (21–23). Another real-life study also supported the utility and safety of S/V in older individuals with HFrEF, while they additionally identified discontinuation of S/V and age as independent predictors of mortality (24). In a cohort of 616 HFrEF patients aged ≥75 years (mean age 83.3), only 14.3% received ARNi therapy despite guideline indications, yet ARNi use was independently associated with a 64% reduction in mortality over 34 months (multivariate HR 0.36, 95% CI: 0.21–0.61), underscoring the need for broader implementation in this under-treated population (25). These insights contribute to the growing body of evidence on the efficacy and challenges of S/V use in older populations, the effectiveness of lower doses, and the importance of continuation in real-world clinical scenarios.

As mentioned, the issue of S/V withdrawal, particularly in older adults, remains a significant concern. One study using US Medicare database found that, among adults above 65 years of age, 65% of them initiated a low dose of S/V(24/26 mg), and 36% of these who were on low doses discontinued within 6 months of initiation (26). Our study aligns with previous investigations, indicating that a substantial proportion of older adults initiate low doses of S/V, with a significant number discontinuing within the following months due to intolerance of S/V (21–24). Comorbidities and polypharmacy are common in the older, leading to drug side effects and interactions. In our cohort, renal dysfunction and hyperkalemia were found to be one of the main reasons for S/V discontinuation. Interestingly, hypotension was less common in older groups which were different from previous studies (3, 4, 24, 27). This might be related to lower initial dose of S/V and gradually titration in the real-world context.

Limited studies have explored the use of S/V in older adults with HFrEF, particularly in clinical practice. Our study found that older heart failure patients were less likely to reach the target dose of ARNi therapy and appeared to benefit less compared to younger patients. This may be due to a greater burden of comorbidities such as chronic kidney disease, hypotension, and atrial fibrillation, which can limit the ability to escalate or maintain ARNi therapy. Age-related physiological decline, polypharmacy, and conservative clinical decision-making may also contribute to suboptimal treatment in this group. Our study is among the first to present real-world data on the Taiwanese population. While current guideline recommendations based on LVEF were consistent across age groups (28), our findings indicate a persistent benefit in achieving at least 50% of the target S/V dose, even in older adults. Therefore, we recommend that physicians aim for a daily dose of at least 200 mg, regardless of age. For patients unable to reach this target, our results reassure the benefits in composite outcomes among younger groups, and a non-significant trend toward reduced risk in older adults. This suggests that partial dosing may still offer protective effects against adverse cardiovascular outcomes compared to discontinuation. Consequently, for patients unable to achieve the target dose, maintaining any level of S/V therapy may be preferable, especially in younger populations as it yields significant better outcomes than cessation.

Our study has some limitations. First, all treatment decisions were based on real-world practice by the participating cardiologists. The lack of universal drug titration and follow-up protocol opens this type of registry to substantial criticisms. However, the current study's objective was to include a broad range of patients reflecting the current reality of clinical practice for S/V and not a specifically defined HF population such as that in clinical trials. Secondly, the adherence to all HF drugs different to S/V was not assessed at the end of follow-up. Thirdly, this survey depends on the hospitals to volunteer their support, introducing bias toward larger centers that can support research staff. Fourth, the ascertainment bias that individuals at better baseline condition might be treated with higher dose S/V should be acknowledged. To address this bias, we performed baselines comparison between treatment results across age groups, which did reveal some differences yet not severe (Supplementary Table S1). In addition, the cox regression model we adopted allowed for the adjustment of these variables, ensuring that these confounding factors did not unduly influence the results. Fifth, our study is the lack of frailty assessment and future research should incorporate validated frailty scores to better capture the real-world challenges of managing older heart failure patients and to guide more personalized treatment strategies with ARNi. Last but not the least, the results in older cohort might be influenced by the small sample sizes, leading to wider confidence intervals and less reliable estimates. Larger studies are needed to confirm these findings and determine the true efficacy of the intervention in older populations.

## 5 Conclusion

In conclusion, our study adds a crucial layer of real-world experience and data to the understanding of S/V use in a cohort of patients with HFrEF in Taiwan. Our findings underscore the need for nuanced treatment strategies. For the younger population, continuing the S/V is still beneficial even if patients don't meet the 50% target; hence, efforts should focus on sustaining participation in the intervention. In the older populations, despite small size and only marginally significant, achieving tolerability is favorable for the trend of benefits. These insights contribute valuable perspectives to the ongoing discourse on the strategy of S/V treatment in real-world practice based on age differences.

## Data Availability

The data supporting this study are derived from the TAROT-HF multicenter registry and are not publicly available due to privacy and ethical restrictions. However, data may be made available upon reasonable request and with appropriate ethical approval and permission from the TAROT-HF registry. Requests should be directed to the corresponding author Dr. Wen-Yu Lin (salmon.lin1019@gmail.com).
