# Supplementary material for: Tolerability for older, persistence for younger: a real-world evidence on sacubitril/valsartan in an Asian heart failure cohort across age
Source: Front Cardiovasc Med. 2025 Jul 22;12:1620266. doi: 10.3389/fcvm.2025.1620266 (PMC12321767; doi:10.3389/fcvm.2025.1620266)
Supplement: Supplementary file 1 [file Table1.docx]

Supplementary Material

# Demographics by age group and ARNi outcome

|  |  | <65 | | | | | | | 65-74 | | | | | | | >=75 | | | | | | |
| --- | --- | --- | --- | --- | --- | --- | --- | --- | --- | --- | --- | --- | --- | --- | --- | --- | --- | --- | --- | --- | --- | --- |
|  |  | DC | | <50% | | >50% | | P | DC | | <50% | | >50% | | P | DC | | <50% | | >50% | | P |
|  |  | N | % | N | % | N | % |  | N | % | N | % | N | % |  | N | % | N | % | N | % |  |
| Gender | Female | 14 | 15.9% | 97 | 20.0% | 81 | 15.2% | .128 | 12 | 21.8% | 60 | 27.8% | 41 | 23.6% | .515 | 25 | 36.8% | 84 | 37.7% | 55 | 37.9% | .986 |
|  | Male | 60 | 68.2% | 325 | 66.9% | 328 | 61.7% |  | 31 | 56.4% | 124 | 57.4% | 87 | 50.0% |  | 38 | 55.9% | 134 | 60.1% | 68 | 46.9% |  |
| DM |  | 28 | 31.8% | 161 | 33.1% | 204 | 38.3% | .166 | 24 | 43.6% | 92 | 42.6% | 87 | 50.0% | .328 | 30 | 44.1% | 89 | 39.9% | 77 | 53.1% | .045 |
| HTN |  | 52 | 59.1% | 190 | 39.1% | 277 | 52.1% | .000 | 30 | 54.5% | 113 | 52.3% | 111 | 63.8% | .069 | 44 | 64.7% | 126 | 56.5% | 98 | 67.6% | .086 |
| Old MI |  | 24 | 27.3% | 112 | 23.0% | 139 | 26.1% | .452 | 24 | 43.6% | 72 | 33.3% | 50 | 28.7% | .119 | 20 | 29.4% | 73 | 32.7% | 47 | 32.4% | .872 |
| Stroke |  | 6 | 6.8% | 42 | 8.6% | 42 | 7.9% | .814 | 6 | 10.9% | 33 | 15.3% | 27 | 15.5% | .681 | 14 | 20.6% | 33 | 14.8% | 13 | 9.0% | .058 |
| Afib |  | 28 | 31.8% | 134 | 27.6% | 122 | 22.9% | .091 | 24 | 43.6% | 79 | 36.6% | 62 | 35.6% | .551 | 30 | 44.1% | 117 | 52.5% | 48 | 33.1% | .001 |
| Dyslipid |  | 32 | 36.4% | 230 | 47.3% | 264 | 49.6% | .069 | 30 | 54.5% | 97 | 44.9% | 79 | 45.4% | .421 | 26 | 38.2% | 90 | 40.4% | 76 | 52.4% | .043 |
| HFH |  | 50 | 56.8% | 279 | 57.4% | 306 | 57.5% | .992 | 36 | 65.5% | 146 | 67.6% | 108 | 62.1% | .523 | 45 | 66.2% | 143 | 64.1% | 96 | 66.2% | .902 |
| NYHA | I-II | 61 | 69.3% | 332 | 68.3% | 361 | 67.9% | .960 | 29 | 52.7% | 152 | 70.4% | 119 | 68.4% | .042 | 43 | 63.2% | 134 | 60.1% | 89 | 61.4% | .892 |
|  | III-IV | 27 | 30.7% | 154 | 31.7% | 171 | 32.1% |  | 26 | 47.3% | 64 | 29.6% | 55 | 31.6% |  | 25 | 36.8% | 89 | 39.9% | 56 | 38.6% |  |
|  |  |  |  |  |  |  |  |  |  |  |  |  |  |  |  |  |  |  |  |  |  |  |
|  |  | Mean | SD | Mean | SD | Mean | SD | P | Mean | SD | Mean | SD | Mean | SD | P | Mean | SD | Mean | SD | Mean | SD | P |
| BMI |  | 25.45 | 4.90 | 26.18 | 4.94 | 27.62 | 5.34 | .000 | 23.66 | 4.32 | 24.33 | 3.93 | 25.21 | 3.91 | .020 | 20.16 | 3.32 | 22.53 | 3.67 | 23.61 | 4.05 | .009 |
| SBP |  | 127 | 20 | 118 | 19 | 125 | 21 | .000 | 120 | 20 | 118 | 18 | 125 | 18 | .001 | 133 | 18 | 115 | 18 | 134 | 27 | .000 |
| eGFR |  | 71.11 | 70.75 | 73.32 | 31.11 | 73.49 | 28.23 | .835 | 45.96 | 27.10 | 59.70 | 25.90 | 58.21 | 23.51 | .001 | 44.09 | 21.00 | 40.99 | 19.00 | 48.38 | 20.32 | .214 |
| LVEF() |  | 29.30 | 6.53 | 26.65 | 6.46 | 27.98 | 6.46 | .000 | 29.45 | 5.82 | 28.12 | 6.64 | 28.58 | 5.76 | .348 | 30.84 | 6.11 | 29.28 | 6.62 | 30.05 | 4.59 | .587 |
| LA dia |  | 44.86 | 8.80 | 47.39 | 9.04 | 46.46 | 8.96 | .052 | 43.66 | 8.40 | 45.39 | 8.88 | 44.19 | 7.84 | .268 | 43.87 | 10.26 | 46.14 | 8.55 | 43.90 | 8.52 | .440 |
| RVSP |  | 38.60 | 14.69 | 38.55 | 15.18 | 37.53 | 14.80 | .579 | 43.37 | 16.60 | 41.23 | 14.46 | 38.26 | 16.13 | .082 | 51.58 | 16.51 | 42.53 | 15.28 | 43.62 | 13.86 | .096 |
|  |  |  |  |  |  |  |  |  |  |  |  |  |  |  |  |  |  |  |  |  |  |  |
| Initial dose |  | 115.91 | 57.38 | 87.53 | 41.84 | 143.73 | 66.57 | .000 | 102.73 | 46.32 | 84.66 | 40.13 | 136.35 | 57.69 | .000 | 96.32 | 47.96 | 84.81 | 41.17 | 132.93 | 51.93 | .000 |
| Final dose |  | 129.55 | 77.45 | 91.62 | 38.10 | 239.47 | 78.73 | .000 | 110.91 | 55.65 | 93.40 | 44.33 | 233.33 | 73.97 | .000 | 95.59 | 50.55 | 83.69 | 31.63 | 239.31 | 79.32 | .000 |
| Total f/u period |  | 809 | 435 | 976 | 380 | 1040 | 322 | .000 | 779 | 409 | 942 | 395 | 1011 | 342 | .000 | 785 | 382 | 822 | 433 | 925 | 379 | .022 |

DC, discontinue

Chi square for category variables, ANOVA for continuous variable
