# Supplementary material for: Tolerability for older, persistence for younger: a real-world evidence on sacubitril/valsartan in an Asian heart failure cohort across age
Source: Front Cardiovasc Med. 2025 Jul 22;12:1620266. doi: 10.3389/fcvm.2025.1620266 (PMC12321767; doi:10.3389/fcvm.2025.1620266)
Supplement: Supplementary file 2 [file Image1.pdf]

A. Primary(Composite) outcome

Variable		N	Events	Pt−Years	Hazard ratio	p
**Age65**						
	Discontinued	81	33	142.4	Reference	
	Below 50%	467	149	1048.0	0.55 (0.37, 0.81)	0.002
	Reach 50%	519	122	1279.4	0.39 (0.26, 0.57)	<0.001
**Age65.74**						
	Discontinued	53	26	76.3	Reference	
	Below 50%	204	69	438.0	0.67 (0.42, 1.09)	0.106
	Reach 50%	166	53	389.0	0.60 (0.37, 0.99)	0.044
**Age75**						
	Discontinued	64	36	92.1	Reference	
	Below 50%	216	101	386.1	0.78 (0.52, 1.15)	0.207
	Reach 50%	141	54	285.2	0.59 (0.38, 0.90)	0.015

0.2 0.5 1 2 5

B. All cause mortaliy

Variable		N	Events	Pt−Years	Hazard ratio	p
**Age65**						
	Discontinued	81	17	182.1	Reference	
	Below 50%	467	68	1241.0	0.47 (0.27, 0.81)	0.006
	Reach 50%	519	41	1476.2	0.28 (0.16, 0.50)	<0.001
**Age65.74**						
	Discontinued	53	18	111.2	Reference	
	Below 50%	204	43	521.3	0.80 (0.44, 1.47)	0.475
	Reach 50%	166	23	460.1	0.53 (0.27, 1.03)	0.060
**Age75**						
	Discontinued	64	19	140.8	Reference	
	Below 50%	216	74	485.0	1.18 (0.70, 1.97)	0.538
	Reach 50%	141	35	355.0	0.86 (0.49, 1.50)	0.589

0.2 0.5 1 2 5

C. Cardiovascular mortality

Variable		N	Events	Pt−Years	Hazard ratio	p
**Age65**						
	Discontinued	81	11	182.1	Reference	
	Below 50%	467	60	1241.0	0.60 (0.31, 1.16)	0.131
	Reach 50%	519	37	1476.2	0.40 (0.20, 0.79)	0.009
**Age65.74**						
	Discontinued	53	13	111.2	Reference	
	Below 50%	204	30	521.3	0.85 (0.41, 1.74)	0.648
	Reach 50%	166	13	460.1	0.48 (0.21, 1.08)	0.077
**Age75**						
	Discontinued	64	11	140.8	Reference	
	Below 50%	216	48	485.0	1.22 (0.63, 2.38)	0.558
	Reach 50%	141	24	355.0	0.99 (0.48, 2.03)	0.984

0.2 0.5 1 2 5

D. First heart failure hospitalization

Variable		N	Events	Pt−Years	Hazard ratio	p
**Age65**						
	Discontinued	81	29	141.5	Reference	
	Below 50%	467	128	1043.8	0.54 (0.35, 0.81)	0.003
	Reach 50%	519	110	1268.7	0.40 (0.26, 0.61)	<0.001
**Age65.74**						
	Discontinued	53	24	75.7	Reference	
	Below 50%	204	55	436.9	0.56 (0.34, 0.93)	0.026
	Reach 50%	166	49	387.9	0.57 (0.34, 0.96)	0.033
**Age75**						
	Discontinued	64	30	92.1	Reference	
	Below 50%	216	84	379.9	0.79 (0.52, 1.22)	0.294
	Reach 50%	141	45	284.1	0.58 (0.37, 0.93)	0.024

0.2 0.5 1 2 5
